# Interaction between diabetes and body mass index on severe headache or migraine in adults: a cross-sectional study

**DOI:** 10.1186/s12877-024-04657-3

**Published:** 2024-01-19

**Authors:** Sheng Tian, Zhijuan Cheng, Heqing Zheng, Xianhui Zhong, Xinping Yu, Jingling Zhang, Lanxiang Wu, Wei Wu

**Affiliations:** 1 Department of Neurology, the Second Affiliated Hospital, Jiangxi Medical College, Nanchang University, No.1, Minde Road, 330006 Nanchang, Jiangxi China; 2 Institute of Neuroscience, Nanchang University, No.1, Minde Road, 330006 Nanchang, Jiangxi China; 3 Department of Anesthesiology and Operative medicine, Medical Center of Anesthesiology and Pain, the First Affiliated Hospital, Jiangxi Medical College, Nanchang University, No.17, 330006 Yongwaizhengjie, Nanchang, Jiangxi China

**Keywords:** Body mass index, Migraine, Diabetes, NHANES, Interactive effects

## Abstract

**Background:**

Research on the effects of body mass index (BMI) on severe headache or migraine is limited and controversial. The aim of this study was to explore the association between BMI and the prevalence of migraine, with particular interest in diabetes status difference.

**Methods:**

The present study used analyzed data from people who participated in the National Health and Nutrition Examination Survey (NHANES) between 1999 and 2004. Logistic regression models and restricted cubic spline (RCS) models were applied to investigate the relationship between body mass index and migraine.

**Results:**

A total of 10,074 adults aged 20 years or older were included in this study. Body mass index was positively related to migraine, and the corresponding odds ratio (OR; 95% CI) was 1.02 (1.01, 1.03; *p* < 0.001). And compared to participants in the lowest group of body mass index (< 25 kg/m^2^), the adjusted ORs for migraine in medium group (25-29.9 kg/m^2^), and highest group (≥ 30 kg/m^2^) were 1.14 (95% CI: 0.98–1.32, *p* = 0.09) and 1.30 (95% CI: 1.11–1.52, *p* = 0.0022), respectively. The relationship between BMI and migraine exhibited a linear in overall in the RCS. Our findings also suggested an interaction between BMI and diabetes. The relationship between BMI and migraine in adults with diabetes was non-linear. The OR of developing migraine was 1.30 (95% CI: 1.10–1.54) in individuals with BMI ≥ 29.71 kg/m^2^ in adults with diabetes.

**Conclusion:**

A higher body mass index is significantly associated with an increased prevalence of migraine, and diabetes status can modify the association between them.

**Supplementary Information:**

The online version contains supplementary material available at 10.1186/s12877-024-04657-3.

## Introduction

Severe headache or migraine is a widespread headache and disabling disorder that has two main subtypes, distinguished by frequency as episodic migraine (< 15 days per month) and chronic migraine (15 or more days per month) lasting for at least 3 months [1]. The prevalence of different types of headaches such as migraine ranges from 7 to 16% in different countries [2]. In particular, migraine is a common source of morbidity in the US population, and its burden is enormous [3]. A growing number of studies have showed gender differences in migraine. It is widely believed that migraine is two to three times more common in women than in men [4, 5]. A study has shown that migraine is the leading cause of disability in adults under the 50 years of age in terms of disability life expectancy [6]. Given the considerable burden that migraine imposes on populations in the United States and elsewhere, understanding the potentially modifiable risk factors for this condition is crucial to helping decrease the morbidity associated with it. In recent years, researchers have examined the possible relationship between obesity and migraine [7, 8]. Because of the rapid increase in obesity rates in the United States, it would be quite meaningful to explore the possible associations between body mass index and migraine.

The results of previous cross-sectional studies regarding the possible association between BMI and migraine have been controversial. In a cross-sectional study of 50,347 Canadian men and women aged 20–64 years, migraine was not observed to be significantly associated with BMI < 18.5 kg/m^2^ or 30 kg/m^2^ [9]. In the 1990s, a study of 1932 American men and women showed that BMI was significantly and positively related to OR value of incident [5.28(1.3–21.1)] and prevalent [1.34(1.0-1.8)] migraine. Also, obese patients with episodic migraine are more likely to transition to chronic daily migraine than patients with normal weight [10]. And diabetes has been reported to be a risk factor in migraine. Therefore, elucidating the relationship between BMI and migraine, as well as the relationship between the two in diabetic populations, may help propose individualized strategies to diminish the negative impact of migraine on public health.

This study examined the relationship between BMI and migraine in adults by utilizing data from NHANES. According to the results found in this database, we hypothesized a positive association between BMI and migraine. Subgroup analyses were conducted to evaluate possible effect modification of the association between BMI and migraine. Furthermore, we also revealed a dose-response relationship between BMI and migraine.

## Methods

### Study participants

This study analyzed data from the NHANES 1999–2004, included by the Centers for Disease Control (CDC) and the National Center for Health Statistics (NCHS). The NHANES is a series of stratified, multi-stage probability surveys for the representative non-institutionalized US civilians to assess the health and nutritional status of adults and children. Demographics, dietary surveys, physical exams, laboratory tests, and other health-related questions are included in NHANES. The study design of NHANES 1999–2004 were approved by the Ethics Review Committee of the National Center for Health Statistics. Methodological details of the NHANES are found at https://www.cdc.gov/nchs/nhanes/index.htm.

**Fig. 1:**
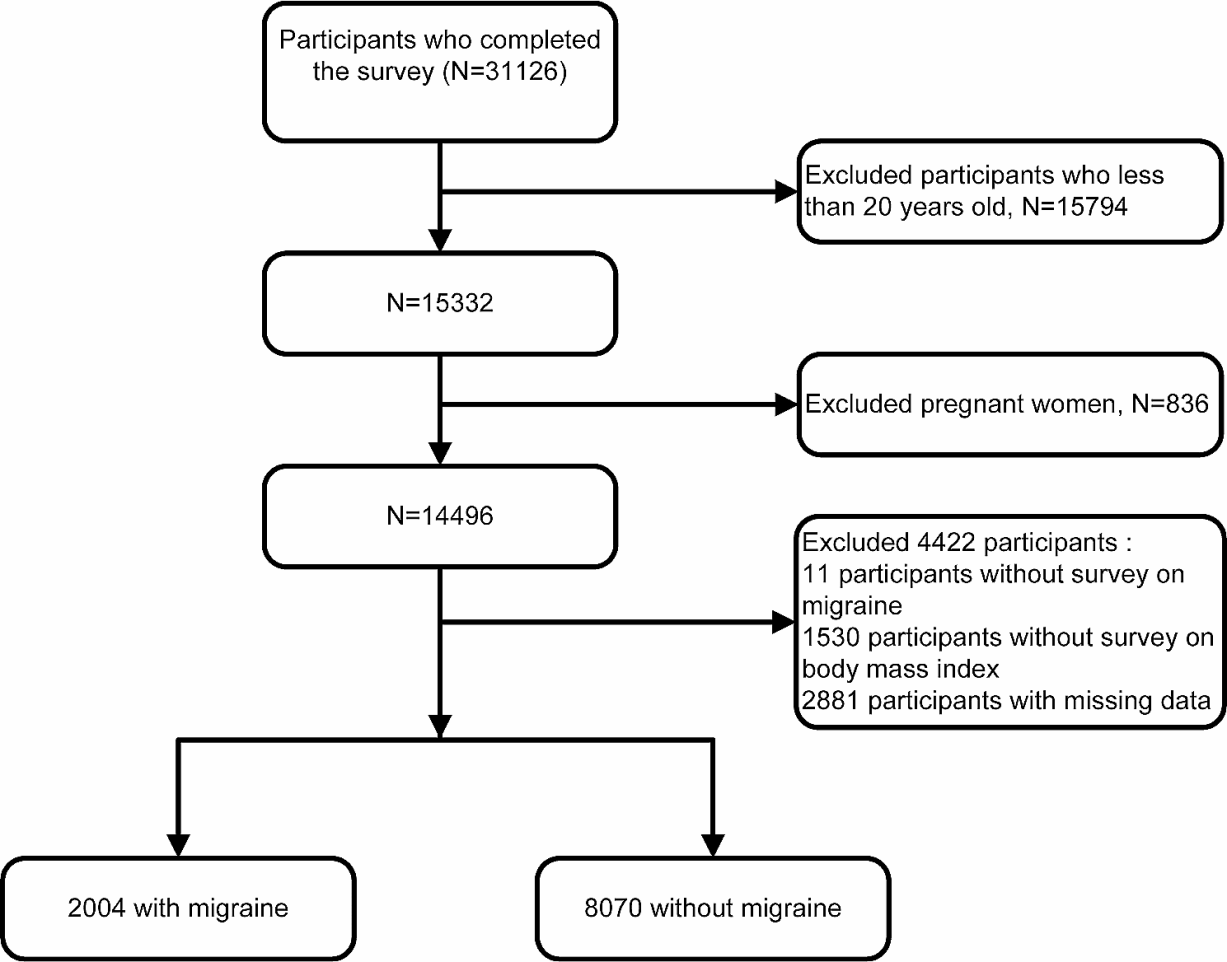
Inclusion and exclusion flow chart

Given that the questionnaire of migraine or severe headache status Questionnaire was only collected between 1999 and 2004, we performed a cross-sectional study in NHANES for those years. These study cycles were combined for our study, which obtained a total of 31,126 participants, and our study was limited to adults 20 years of age or older. We excluded pregnant women and individuals with missing values, such as migraine questionnaires and BMI data. In the end, 10,074 participants included in this study (Fig. 1).

### Migraine classification

The identification of migraine was assessed based on the self-reported to the NHANES Miscellaneous Pain Questionnaire (MPQ), where question MPQ090 asked, “In the past three months, did you have severe headaches or migraines?” We divided participants who answered ‘yes’ into those with migraine. And we can consider that the majority of participants with severe headache has migraine, which is consistent with the results of the American Migraine Prevalence and Prevention (AMPP) study. The study showed that 17.4% of individuals reported “severe headache”, of which 11.8% met the International Headache Disorder Type II (ICHDII) criteria for migraine, 4.6% met the criteria for “probable migraine”, and only 1% were identified as “other severe headache” [11].

### Body mass index assessment and potential covariates

In the mobile examination center (MEC), a physical examination of the individual was performed to obtain information about the participant’s BMI, including height, weight. BMI is calculated by dividing weight (kg) by the square of height (m), rounded to two decimal places. According to the classification reported in previous literature, BMI was classified into three categories: <25.0 kg/m^2^, 25.0-29.9 kg/m^2^ and ≥ 30.0 kg/m^2^ [12, 13]. Various potential covariates were included based on the exited literature [14–17]. Demographic covariates included age, sex, race, marital status (married, living alone), education level (< high school, high school, >high school), and family income, which were obtained by self-report during interviews with the researcher [18, 19]. The poverty income ratio (PIR) divided family income into three categories: low (PIR ≤ 1.3), medium (PIR > 1.3 ~ 3.5) and high (PIR > 3.5) [20]. C-reactive protein (CRP) was quantified through latex-enhanced nephelometry. According to previous descriptions in the literature, drinking and smoking status was classified as never, current and former. History of stroke, hypertension, coronary heart disease or diabetes was also evaluated as self-reported physician diagnosis of stroke, hypertension, coronary heart disease or diabetes [21].

### Statistical analyses

No statistical power calculations were performed before data analysis, and the sample size was all public data from NHANES. MEC weights, primary sampling units and strata information were exerted in the statistical analysis. For the joint analysis of the NHANES 1999–2000 and 2001–2002 data, we used the 4-year MEC weight (WTMEC4YR) set. For the 2003–2004 data, we used the 2-year MEC exam weight (WTMEC2YR) set. According to the analytical guidelines found on the NHANES website, we calculated sampling weights for 1999–2004 = 2/3 of the 1999–2002 weight or 1/3 of the 2003–2004 weight [22]. Continuous variables were described by sample-weighted means (standard error, SE), while categorical variables were reported as sample-weighted percentages and frequencies. To compare the differences between different groups, One-way analyses of variance (continuous variables) and Chi square tests (categorical variables) were conducted. Multiple logistic regression models were used to evaluate odd ratios (ORs) with 95% confidence intervals (CIs) of migraine at different groups of body mass index before or after adjustment of confounders. The lowest group of body mass index was considered as the reference group. Next, potential modifications of the relationship between body mass index and migraine were evaluated for the following variables: diabetes, sex, age (20–50, > 50years).

Furthermore, restricted cubic spline (RCS) regression was applied to examine the nonlinear relationship between body mass index and migraine. Body mass index was taken as continuous variables to be included in the model. After adjusting for all potential confounding variables, we used a two-piece-wise logistic regression model with smoothing to explore the threshold for the association between dietary selenium intake and migraine. Inflection points were identified by using likelihood-ratio test and the bootstrap resampling method. All statistical analysis was conducted by using R software (version 4.2.1). Two-sided *p* values < 0.05 was considered statistically significant difference.

## Results

### Baseline characteristic

Supplementary materials (Table S1) showed the general characteristics of the included and excluded participants. The general characteristics of the 10,074 included participants based on different groups of body mass index are demonstrated in Table 1. A total of 2004 participants with migraine, while 8070 participants without migraine. The average age of the participants in this study was 46.36(0.30) years, and 4941 (50.58) participants were female. Individuals who had higher BMI tended to be older, female, non-Hispanic White, married, had a higher educational level, had a high family income, never smoking, current drinking, had a higher incidence of hypertension, stroke, coronary heart disease and diabetes, migraine and higher serum CRP levels. As shown in Table 2, the findings of the univariate analysis demonstrated that age, sex, race, education level, family income, smoking status, drinking status, diabetes and C-reactive protein were related to migraine.

**Table 1:** Population characteristics by categories of body mass index

Characteristic^a^	Body mass index, kg/m^2^				
	**Total**	< 25.0	25.0-29.9	≥ 30	*P* value ^b^
**No.**	**10,074**	**3181**	**3657**	**3236**	
Age, years, mean (SE)	46.36(0.30)	43.65(0.39)	48.31(0.40)	47.20(0.40)	< 0.0001
Sex, n (%)					< 0.0001
Male	5133(49.42)	1543 (43.59)	2137 (58.19)	1453 (46.08)	
Female	4941(50.58)	1638 (56.41)	1520 (41.81)	1783 (53.92)	
Marital status, n (%)					< 0.0001
Living alone	3766(34.50)	1359 (39.55)	1203 (30.12)	1204 (33.79)	
Married	6308(65.50)	1822 (60.45)	2454 (69.88)	2032 (66.21)	
Race, n (%)					< 0.0001
Non-Hispanic White	5326(74.17)	1876 (77.01)	1893 (73.54)	1557 (71.73)	
Non-Hispanic Black	1835 (9.67)	492(7.78)	581 (8.39)	762 (13.20)	
Mexican American	2176 (6.72)	543 (5.36)	902 (7.69)	731 (7.69)	
Others	737 (9.44)	270 (9.86)	281 (10.39)	186 (7.91)	
Education level, n (%)					< 0.001
<High school	3073(18.72)	859 (16.90)	1201 (19.56)	1013 (19.79)	
High school	2393(26.00)	728 (23.72)	840 (26.16)	825 (28.36)	
>High school	4608(55.28)	1594 (59.38)	1616 (54.28)	1398 (51.84)	
Family income, n (%)					< 0.001
Low	2761(20.46)	877 (21.48)	944 (17.56)	940 (22.58)	
Medium	3904(35.85)	1194 (34.11)	1430 (35.96)	1280 (37.65)	
High	3409(43.69)	1110 (44.41)	1283 (46.48)	1016 (39.77)	
Smoking status, n (%)					< 0.0001
Never	5044(49.67)	1579 (49.56)	1801 (48.51)	1664 (51.08)	
Current	2258(24.92)	898 (29.96)	714 (22.47)	646 (22.08)	
Former	2772(25.41)	704 (20.48)	1142 (29.02)	926 (26.84)	
Drinking, n (%)					< 0.0001
Never	1425(12.13)	427 (11.62)	499 (11.05)	499 (13.90)	
Current	6565(70.75)	2220 (76.27)	2417 (72.12)	1928 (63.10)	
Former	2084(17.12)	534 (12.11)	741 (16.83)	809 (22.99)	
Diabetes, n (%)	1020 (6.73)	169 (3.26)	366 (5.88)	485 (11.52)	< 0.0001
Hypertension, n (%)	3326(27.77)	659 (15.46)	1202 (27.89)	1465 (41.31)	< 0.0001
Stroke, n (%)	331 (2.31)	82 (1.60)	129 (2.55)	120 (2.84)	0.003
Coronary heart disease, n (%)	483 (3.74)	116 (2.37)	190 (4.35)	177 (4.58)	< 0.0001
C-reactive protein, mg/dl, mean (SE)	0.41(0.01)	0.27(0.01)	0.38(0.01)	0.62(0.02)	< 0.0001
Migraine, n (%)	2004(21.54)	620 (20.48)	658 (19.40)	726 (25.12)	< 0.0001

^a^ mean and percentages were weighted. ^b^*p* value was calculated by weighted one-way analyses of variance for continuous variable and Chi-square test for categorical variables

**Table 2:** Relationship of covariates and migraine risk

Variable	OR (95% CI)	*P* value
Age (years)	0.97(0.97,0.98)	< 0.0001
Sex		
Male	Reference	
Female	2.04(1.81,2.30)	< 0.0001
Marital status		
Living alone	Reference	
Married	0.95(0.85,1.06)	0.34
Race		
Non-Hispanic White	Reference	
Non-Hispanic Black	1.28(1.07,1.52)	0.008
Mexican American	1.16(0.99,1.37)	0.07
Others	1.30(0.97,1.74)	0.08
Education level		
<High school	Reference	
High school	0.85(0.74,0.98)	0.03
>High school	0.68(0.58,0.79)	< 0.0001
Family income		
Low	Reference	
Medium	0.68(0.57,0.82)	< 0.0001
High	0.47(0.40,0.57)	< 0.0001
Smoking status		
Never	Reference	
Current	1.34(1.15,1.57)	< 0.001
Former	0.77(0.66,0.90)	0.001
Drinking		
Never	Reference	
Current	0.82(0.71,0.96)	0.01
Former	1.03(0.83,1.30)	0.76
Diabetes		
no	Reference	
yes	1.41(1.01,2.02)	0.04
Hypertension		
no	Reference	
yes	1.07(0.96,1.18)	0.23
Stroke		
no	Reference	
yes	0.91(0.76,1.08)	0.27
Coronary heart disease		
no	Reference	
yes	0.71(0.49,1.04)	0.08
C-reactive protein(mg/dl)	1.10(1.04,1.17)	0.001

Data were present as odds ratio (OR) and 95% confidence interval (CI)

### Association between BMI and migraine

The findings of the multi-factor logistic regression models were shown in Table 3. In the crude, body mass index was positively associated with migraine. After adjustment by age and sex, the findings of model 1 were generally consistent with the crude. In the model 2, body mass index was positively associated with migraine, and the corresponding odds ratio (OR; 95% CI) was 1.02 (1.01, 1.03; *p* < 0.001). And compared to individuals in the lowest group of body mass index (< 25 kg/m^2^), the adjusted ORs for migraine in medium group (25-29.9 kg/m^2^), and highest group (≥ 30 kg/m^2^) were 1.14 (95% CI: 0.98–1.32, *p* = 0.09) and 1.30 (95% CI: 1.11–1.52, *p* = 0.0022), respectively. In the RCS analyses (Figure S1), we found a linear association between body mass index and migraine (*P* > 0.05). The OR values for the association between BMI and migraine were elevated with increasing BMI.

**Table 3:** Association between body mass index and migraine

BMI (kg/m^2^)	OR (95% CI)						
	No.	**Crude**	*P* value	Model 1	*P* value	Model 2	*P* value
Continuous	10074	1.02(1.01,1.03)	< 0.0001	1.03(1.02,1.03)	< 0.0001	1.02(1.01,1.03)	< 0.001
Lowest (< 25)	3181	Reference		Reference		Reference	
Medium (25-29.9)	3657	0.94(0.81,1.08)	0.35	1.17(1.02,1.35)	0.03	1.14(0.98,1.32)	0.09
Highest (≥ 30)	3236	1.30(1.14,1.50)	< 0.001	1.47(1.29,1.69)	< 0.0001	1.30(1.11,1.52)	0.0022
P for trend	-	< 0.001	-	< 0.001	-	0.002	-

Crude was adjusted by nothing. Model 1 was adjusted for age and sex. Model 2 was adjusted for Model 1 + marital status, race, education level, family income, smoking status, drinking, hypertension, coronary heart disease, stroke, diabetes, and C-reactive protein. BMI Body mass index, OR Odds ratio, CI Confidence interval

### Subgroup analysis

**Fig. 2:**
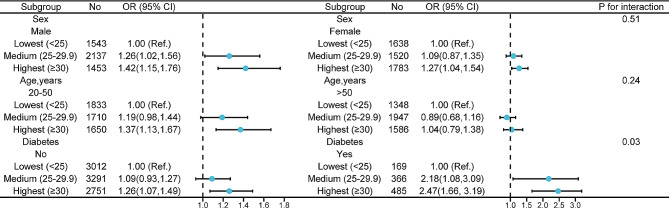
Effect of body mass index on migraine in different subgroup (sex, age, diabetes). Except the stratification variables themselves, each stratification factor was adjusted for all other variables (sex, age, marital status, race, education level, family income, smoking status, drinking, hypertension, coronary heart disease, stroke, diabetes, and C-reactive protein)

Subgroup analysis were performed to explore the possible effect modifications of the association between body mass index and migraine. After stratification by diabetes, sex and age, a significant interaction was observed between body mass index and diabetes (Fig. 2). The multivariate logistic regression model exhibited that the adjusted ORs for migraine were 2.18 (95% CI: 1.08–3.09) and 2.47 (95% CI: 1.66–3.19), respectively. Additionally, in the participants without diabetes group, we observed that body mass index was also related to the prevalence of migraine, but the OR value appeared to be smaller. Finally, although the positive correlation between body mass index and migraine seemed to be stronger among male and among participants with 20–50 years old, the interactions were not statistically significant (p for interaction = 0.51, 0.24, respectively) (Fig. 2).

**Fig. 3:**
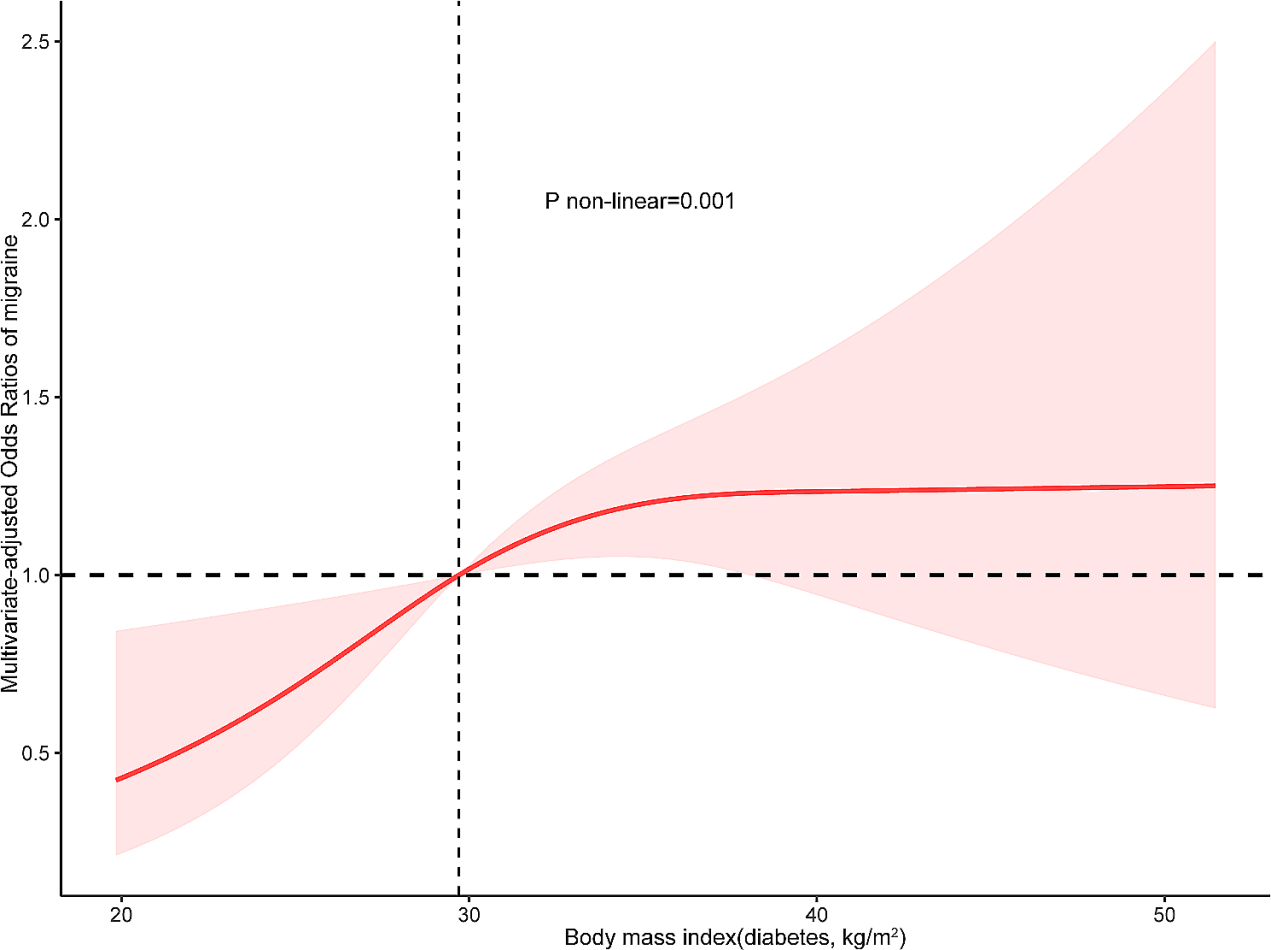
Association between body mass index and migraine odds ratio in diabetes group. The model was adjusted for sex, age, marital status, race, education level, family income, smoking status, drinking, hypertension, coronary heart disease, stroke and C-reactive protein

In the Fig. 3, the restricted cubic spline revealed that a non-linear relationship between body mass index and migraine in the diabetes group (*P* = 0.001), using a reference point of 29.71 kg/m^2^. Also, in the threshold analysis, the OR for migraine was 1.30 (95% CI: 1.10–1.54, *p* = 0.003) in individuals with a BMI of ≥ 29.71 kg/m^2^ (Table 4). It means that the risk of migraine raised with increasing BMI. There was no relationship between body mass index and migraine when the body mass index<29.71 kg/m^2^ (Table 4). It means that the risk of migraine no longer raised with increasing body mass index.

**Table 4:** Threshold effect analysis of the association of body mass index with migraine in adults with diabetes

Body mass index kg/m^2^	Adjust OR (95% CI)	*P* value
< 29.71	1.04(0.99,1.09)	0.11
≥ 29.71	1.30(1.10,1.54)	0.003
Log-likelihood ratio test		0.02

Data were present as odds ratio (OR) and 95% confidence interval (CI). Fully adjusted for sex, age, marital status, race, education level, family income, smoking status, drinking, hypertension, coronary heart disease, stroke and C-reactive protein

## Discussion

In present cross-sectional study, we offered the nationally representative evidence of the association between body mass index and migraine in American adults. In this nationally representative cross-sectional study, we found a positive relationship between body mass index and migraine. The results were not modified by sex, by age. However, diabetes can modify the relationship between body mass index and migraine. Particularly, the increased body mass index was related to a significantly higher risk of migraine in the diabetes group, but this positive relationship between body mass index and migraine seemed to be smaller in without diabetes group. And we found a non-linear association between body mass index and migraine in the diabetes group (*P* = 0.001).

The effect of BMI on migraine has only been descripted in a few studies. An observational study in Iran observed that migraineurs with higher BMI experienced higher headache frequency, severity and duration as well as higher disability score [23]. A case-control study in China found a significant difference between BMI and migraine attack frequency and that migraineurs were tended to be overweight, obese or morbidly obese compared to the healthy group [24]. A recent meta-analysis found that obese individuals had a significantly increased risk of migraine compared to individuals with a normal BMI. Also, there was a non-linear relationship between BMI and migraine, with an elevated risk at BMI values > 29 [25]. It is noteworthy that all of the researches above studies were mostly case-control studies or case series, and no additional study has been conducted to evaluate the association between BMI and migraine in the general population. Therefore, NHANES affords us the excellent chance to verify the existence of a positive association between BMI and migraine, which will provide a basis for further determination of the effect of BMI on migraine.

The results of our nationally representative study provide new insights. Firstly, this study offered epidemiological evidence of a significant association between body mass index and migraine in a general population across the United States, compared to previous studies with small samples. Secondly, we observed that there was an interaction between diabetes and body mass index, and that diabetes can modify the association between body mass index and migraine (P for interaction = 0.03). Body mass index was significantly associated with an increased incidence of migraine in individuals with diabetes, but positive relationship between them appeared to be smaller in participants without diabetes. Leptin is an important adipose tissue-derived hormone that is involved in the pathophysiological mechanisms of diabetes [26]. It has been shown that serum leptin levels are significantly elevated in obese diabetic patients and it is believed that elevated serum leptin levels are associated with an increased risk of diabetes [27, 28]. A study revealed that in obese individuals, serum leptin levels were higher and positively correlated with BMI [29]. Furthermore, elevated leptin level is thought to induce the secretion of pro-inflammatory factors (IL-6 and TNF- α) and NO that play a role in migraine through the NF-κβ signaling pathway [30, 31]. And the current findings found that administration of leptin to Wistar rats could diminish the threshold of pain [32]. Thus, participants with diabetes may have higher levels of serum leptin than those without diabetes, which may explain why BMI is more significantly associated with migraine in participants with diabetes. And in the present study, the results of RCS showed that ORs increased significantly (> 1.00) individuals with diabetes, when BMI was greater than 29.71 kg/m^2^. This result was supported by the threshold effect analysis. Therefore, the positive association between body mass index and migraine may be stronger in obese diabetic individuals. In sum, the positive association between BMI and migraine in individuals with diabetes is important to propose an individualized strategy for migraine prevention in adults.

Although the underlying mechanism of the positive relationship between BMI and migraine is unclear, it seems reasonable in terms of biology. Obesity is a low-grade inflammation that is presented by elevated concentrations of circulating cytokines such as C-reactive protein, tumor necrosis factor-α) and interleukins such as IL-6 [33]. Inflammation has been perceived as a possible causal mechanism for migraine [34, 35]. Moreover, concentrations of calcitonin gene-related peptide (CGRP) were elevated in the obese individuals and were higher in patients with migraine and other headaches type than in those without headaches symptoms [36, 37]. A study has found that neuropeptides and their receptors are the main important targets for migraine treatment due to the important role of CGRP in the pathogenesis of migraine [38]. Also, it has been proposed that administration of CGRP induces the accumulation of fat in an animal model of obesity [39]. And substance P (SP), another factor that may play a role in migraine attack pathogenesis, has also been observed in adipose tissue and exerts a role in the onset of fat accumulation and obesity-associated inflammatory cascade [40]. Thus, these findings showed that the inflammatory properties of obesity (BMI ≥ 30 kg/m^2^) may contribute to its adverse effects on migraine.

There are some limitations that must be taken into account in this study. First of all, because of the population observed in this study was American and did not include special populations, we were unable to analyze special individuals or other races due to the limited sample size. Thus, the general applicability of these results remains to be examined. In addition, the identification of migraine was limited to a single question of migraine or severe headache within the past 3 months. Therefore, it is quite difficult to analyze the different subgroups of migraine separately. Nonetheless, previous studies have supported the consistency of this migraine assessment with criteria for migraine and possible migraine, as well as others who have published articles on migraine with this dataset on the basis of supporting data from the American Migraine Prevalence and Prevention Study [11, 41]. Therefore, these data provide meaningful insight into the otherwise lack of epidemiologic data linking BMI and migraine. Secondly, Data specific to type of diabetes were not included in the NHANES data, which is consistent with previous studies [42, 43]. As a result, it is not possible to distinguish the types of diabetes. And we cannot eliminate the effect of nonrandom missing data on the results, because of baseline differences between included and excluded participants. Third, we cannot exclude the possibility that the observed relationships are due to unmeasured confounders, although we have adjusted for many confounding variables. Finally, the present study was a cross-sectional study, which meant that causal inferences cannot be made. Therefore, we will need to conduct the prospective cohort study to obtain more accurate evidence.

This study presented several novelties and advantages. At first, this study provided epidemiological evidence of the significant association between body mass index on migraine in a representative general population across the United States. Secondly, with the adjustments for potential confounding factors, our conclusions are more realistic. And, subgroup analyses were performed in several subgroups to identify any existing differences. Furthermore, we evaluated the dose-response effect of body mass index on migraine and provided practical recommendations. Third, studies on the positive association between body mass index and migraine are relatively rare. In addition, no previous studies on the interaction between body mass index and diabetes on migraine have been published. This study indicated that reducing body mass index may help prevent migraine.

## Conclusions

In conclusion, we found a positive relationship between body mass index and severe headache or migraine, and the association was non-linear in the diabetes group. Especially, the positive correlation between body mass index and severe headache or migraine is stronger in individuals with the diabetes than in those without diabetes. Therefore, improving diabetes status may alter the effect of body mass index on migraine, which is significant for proposing individualized prevention strategies for migraine.

### Electronic supplementary material

Below is the link to the electronic supplementary material.

**Supplementary Material 1: Table S1.** Basic characteristics of excluded and included participants

**Supplementary Material 2: Figure S1.** Association between body mass index and migraine odds ratio in overall. The model was adjusted for sex, age, marital status, race, education level, family income, smoking status, drinking, hypertension, coronary heart disease, stroke, diabetes and C-reactive protein

## Data Availability

This study analyses publicly available data. The raw data can be found in the repository here: https://www.cdc.gov/nchs/nhanes/index.htm.

## Abbreviations

AMPM: Automated Multiple Pass Method
AMPP: American Migraine Prevalence and Prevention
BMI: Body mass index
CRP: C-reactive protein
ICHDII: International Headache Disorder Type II
MPQ: Miscellaneous Pain Questionnaire
NCHS: National Center for Health Statistics
NHANES: National Health and Nutritional Examination Survey
PIR: Poverty income ratio
RCS: Restricted cubic spline
USDA: United States Department of Agriculture
