# Supplementary material for: Interaction between diabetes and body mass index on severe headache or migraine in adults: a cross-sectional study
Source: BMC Geriatr. 2024 Jan 19;24:76. doi: 10.1186/s12877-024-04657-3 (PMC10799418; doi:10.1186/s12877-024-04657-3)
Supplement: Supplementary file 1 — Supplementary Material 1: Table S1. Basic characteristics of excluded and included participants [file 12877_2024_4657_MOESM1_ESM.docx]

**Table S1.** Basic characteristics of excluded and included participants.

|  | Excluded population | Included population | *P* value ^b^ |
| --- | --- | --- | --- |
| Number of subjects ^a^ (%) | 21052(67.6%) | 10074 (32.4%) |  |
| Sex (%) |  |  | <0.001 |
| Male | 10051 (47.7) | 5133 (51.0) |  |
| Female | 11001 (52.3) | 4941 (49.0) |  |
| Age(year) | 19.56 (21.11) | 50.80 (18.51) | <0.001 |
| Race (%) |  |  |  |
| Non-Hispanic White | 6780 (32.2) | 5326 (52.9) | <0.001 |
| Non-Hispanic Black | 5737 (27.3) | 1835 (18.2) |  |
| Mexican American | 6512 (30.9) | 2176 (21.6) |  |
| Others | 2023 (9.6) | 737 (7.3) |  |
| Marital status (%) |  |  |  |
| Living alone | 7115 (71.1) | 3766 (37.4) | <0.001 |
| Married | 2893 (28.9) | 6308 (62.6) |  |
| Education (%) |  |  |  |
| <High school | 11617 (73.6) | 3073 (30.5) | <0.001 |
| High school | 1831 (11.6) | 2393 (23.8) |  |
| >High school | 2338 (14.8) | 4608 (45.7) |  |
| Family income (%) |  |  |  |
| Low | 8019 (44.1) | 2761 (27.4) | <0.001 |
| Medium | 6413 (35.3) | 3904 (38.8) |  |
| High | 3756 (20.7) | 3409 (33.8) |  |
| Smoking status |  |  |  |
| Never | 2861 (54.8) | 5044 (50.1) | <0.001 |
| Current | 1045 (20.0) | 2258 (22.4) |  |
| Former | 1317 (25.2) | 2772 (27.5) |  |
| Drinking |  |  |  |
| Never | 569 (18.9) | 1425 (14.1) | <0.001 |
| Current | 1763 (58.6) | 6565 (65.2) |  |
| Former | 674 (22.4) | 2084 (20.7) |  |
| Diabetes | 556 (2.8) | 1020 (10.1) | <0.001 |
| Hypertension | 1688 (19.8) | 3326 (33.0) | <0.001 |
| Stroke | 275 (5.3) | 331 (3.3) | <0.001 |
| Coronary heart disease | 224 (4.3) | 483 (4.8) | 0.23 |
| C-reactive protein (mg/dl) | 0.28 (0.69) | 0.47 (0.93) | <0.001 |
| Migraine | 1041 (19.8) | 2004 (19.9) | 0.96 |

^a^ mean and percentages are unweighted

^b^ p value was calculated by independent t-test for continuous variable and Chi-square test for categorical variables

BMI body mass index
