# Supplementary material for: Interaction between diabetes and body mass index on severe headache or migraine in adults: a cross-sectional study
Source: BMC Geriatr. 2024 Jan 19;24:76. doi: 10.1186/s12877-024-04657-3 (PMC10799418; doi:10.1186/s12877-024-04657-3)
Supplement: Supplementary file 2 — Supplementary Material 2: Figure S1. Association between body mass index and migraine odds ratio in overall. The model was adjusted for sex, age, marital status, race, education level, family income, smoking status, drinking, hypertension, coronary heart disease, stroke, diabetes and C-reactive protein [file 12877_2024_4657_MOESM2_ESM.docx]

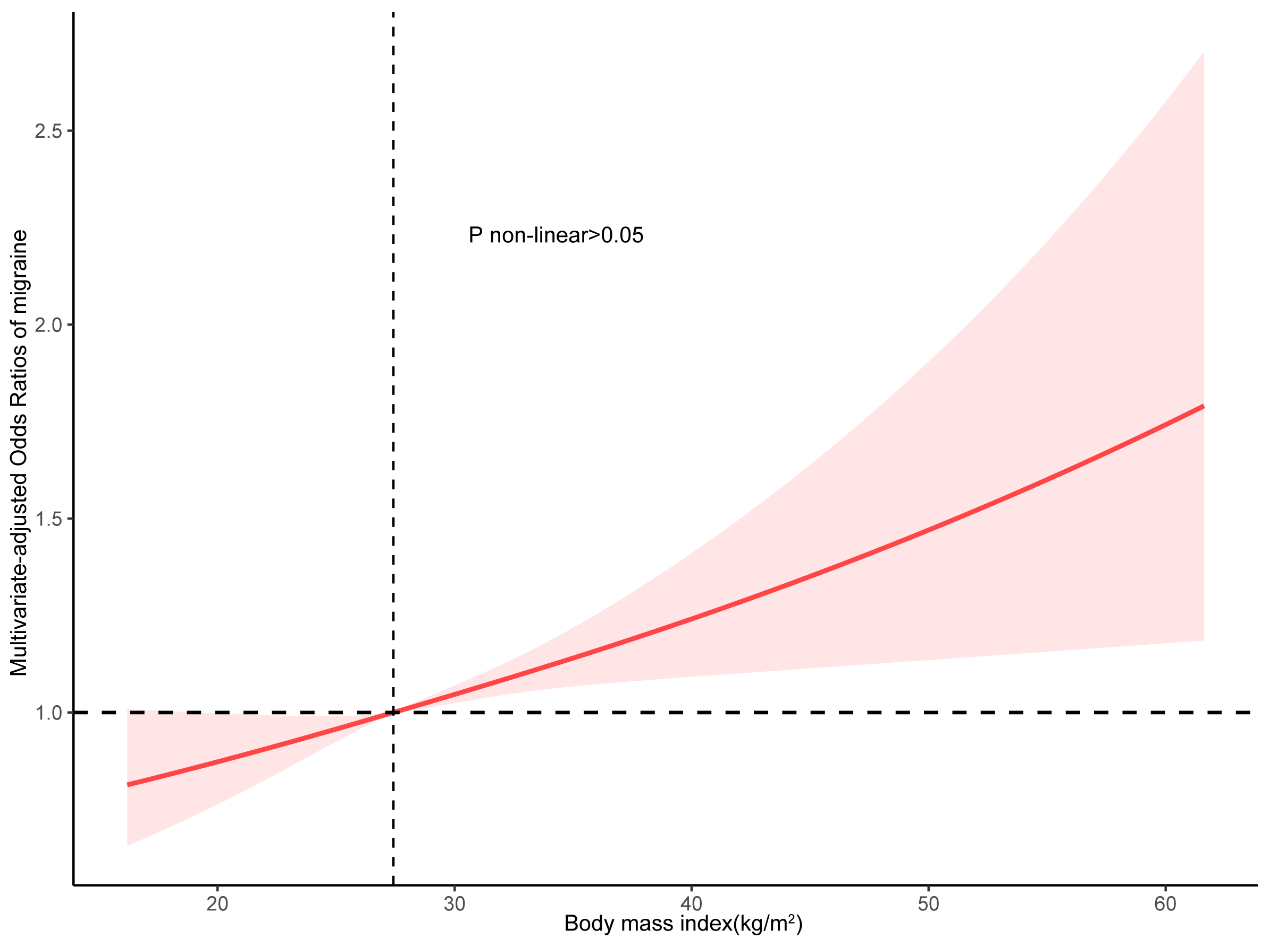


**Figure S1.** Association between body mass index and migraine odds ratio in overall. The model was adjusted for sex, age, marital status, race, education level, family income, smoking status, drinking, hypertension, coronary heart disease, stroke, diabetes and C-reactive protein.
